# Construction and functional verification of size-reduced plasmids based on TMP resistance gene *dfrB10*

**DOI:** 10.1128/spectrum.01206-23

**Published:** 2023-10-31

**Authors:** Chun-cao Li, Rui Hu, Xiu-min Hua, Yi-xuan Ni, Lu Ge, Lu Zhang, Wen Yu, Ni-xin Hao, Hui Xia, Qiang Fang, Zhi-yong Tao

**Affiliations:** 1 Department of Microbiology and Parasitology, Bengbu Medical College, Bengbu, Anhui, China; 2 Anhui Key Laboratory of Infection and Immunology, Bengbu Medical College, Bengbu, Anhui, China; University of Mississippi, University, Mississippi, USA

**Keywords:** TMP, *dfrB10*, drug selection marker, plasmid, vector

## Abstract

Antibiotic resistance screening is crucial in the construction of recombinant plasmids. The *dfrB10* gene, a short-size trimethoprim (TMP)-resistance type B dihydrofolate reductase gene, is a promising antibiotic drug screening marker for plasmid construction in molecular genetic research. Currently, available plasmid backbones (without the resistance gene) were amplified using PCR, and then we ligated these products with *dfrB10* gene fragments via homologous recombination to construct new plasmid vectors with TMP resistance. The cloning, expression, and gene editing functions of the new plasmids based on their different purposes were verified. By successfully replacing the original resistance genes of pUC19, pET28a, pcDNA3.1, and pX330 plasmids with *dfrB10*, we constructed a series of plasmids with TMP resistance. The change of the resistance selection marker to TMP did not affect the function of the plasmids compared to the original ones. Furthermore, a short (988 bp) TMP-resistant miniature tool plasmid, pTMi, was constructed based on the pUC19 plasmid. With the use of the *dfrB10* gene as a resistance marker under TMP drug screening, various size-reduced tool plasmids can be routinely constructed. These plasmids have similar functions to the original ones and can be used for research applications in molecular genetics.

**IMPORTANCE:**

Plasmid size is one of the factors affecting transfection efficacy in most of the molecular genetic research studies. One effective approach for reducing plasmid size is to replace relatively large, conventional antibiotic resistance genes with the short-size *dfrB10* gene. The successful construct of a series of *dfrB10-*based tool plasmids and their functional validation, via comparison with original plasmids, suggest that *dfrB10* is a potent drug resistance selection marker. The antibiotic trimethoprim offers convenient usage comparable to that of ampicillin or kanamycin. Additionally, fluorescence analysis has demonstrated the compatibility of TMP with protein expression in various host cells. Based on these findings, TMP-*dfrB10* could be an alternative choice for future use in molecular genetic research studies that require miniature plasmids to achieve optimal results.

## INTRODUCTION

Plasmids are one of the most commonly used tools for recombinant gene expression and genetic engineering (1, 2). The functions of plasmids depend on the different element parts on their backbone, and plasmid size often impacts their effectiveness in molecular genetic research (3). Natural plasmids come in varying sizes, ranging from less than 1 kb to over 100 kb. Researchers typically use smaller plasmids because they are easier to isolate, manipulate, and sequence (4). More importantly, plasmid size impacts the efficiency of gene transfer with smaller plasmids being more amenable to experiments such as transformation and transfection. Miniature plasmid vectors have been found to be very effective for mammalian cell transfection and *in vivo* gene therapy (5). Mesenchymal stem cells were transfected with large- or small-sized plasmid, either at equimolar or equimass concentrations, and the transfection efficiency is directly linked to the physical size of plasmid, a larger plasmid being more toxic and harder to transfect than a small plasmid (6). Recent research found that using the minicircle or nanoplasmid technique could further reduce or remove bacterial-derived elements on the constructs and increase the efficiency of transfection and expression (7, 8). However, modifying the currently available plasmids to reduce the size is a feasible and practical way.

Size-reduced plasmids are of great interest in molecular biology. Plasmid miniaturization can be achieved either through direct synthesis or through modification of an existing plasmid (9). The inducible plasmid pminiR1 containing minimal elements is a successful example of using synthetic technology to construct a new plasmid. *Escherichia coli* (*E. coli*) transformed with pminiR1 can be induced to increase the copy number (10). One of the smallest reported tool plasmids is pICOz, which is 1,185 bp long and is based on pUC18 with a shortened antibiotic screening maker (5). Usually, it is necessary to use antibiotics to screen positive clones after the plasmid transformation of bacteria. Most common antibiotic resistance genes include ampicillin (AMP), kanamycin (KANA), and so on. However, the length of these antibiotic resistance genes is relatively long, usually more than 800 bp, and shortening their length is one of the ways to miniaturize the plasmid.

Trimethoprim is a competitive inhibitor of dihydrofolate reductase (DHFR), an enzyme in the bacterial folate metabolism pathway, and has been used since 1960s to treat bacterial infections. TMP is effective against a broad spectrum of bacteria (11), including *E. coli* that is a commonly used bacterium in molecular cloning. However, with the extensive use of TMP, bacteria have developed resistance to it (12, 13). Bacteria can resist TMP by expressing the *dfr* gene, which encodes for dihydrofolate reductase. There are two types of TMP resistance genes: dihydrofolate reductase A and B (*drfA* and *dfrB*) (14, 15). Compared to *dfrA*, the *dfrB* family genes have a smaller size, and *dfrB10* is only 237 bp long. The *dfrB10* encodes a 78-amino-acid protein that forms a tetramer with dihydrofolate reductase function (16). This study investigated the use of *dfrB10* as a resistance gene in tool plasmids and the replacement of larger resistance genes like AMP and KANA with *dfrB10*. The plasmid size was reduced by about 0.5 kb while maintaining resistance to TMP.

## RESULTS

### Functional validation of *dfrB10* gene against antibiotic TMP

**Fig 1:**
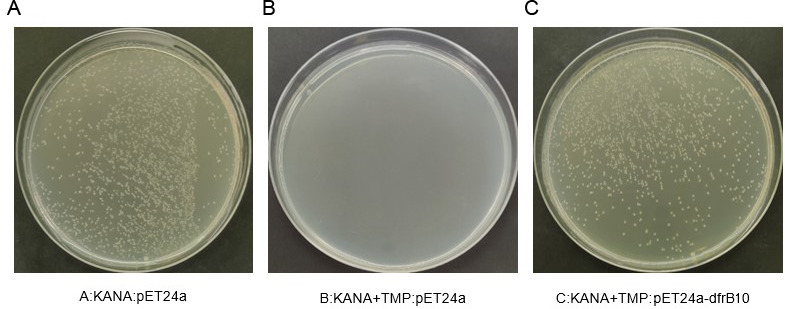
Growth of recombinant pET24a-*dfrB10 E. coli* on a TMP plate. (**A**) Positive control: *E. coli* transformed with empty pET24a growing on a plate containing only KANA antibiotic. (**B**) Negative control: empty *E. coli* BL21 DE3+ could not grow on the plate containing both KANA and TMP antibiotics. (**C**) Recombinant pET24a-*dfrB10*-transformed *E. coli* growing well on the plate containing both KANA and TMP antibiotics, colonies similar to positive control.

After the *dfrB10* gene was synthesized and inserted into the pET24a vector, the sequencing results confirmed that the sequence of pET24a-*dfrB10* plasmid was consistent with the design. The transformed pET24a-*dfrB10* plasmid conferred TMP resistance to *E. coli* strain BL21 DE3+ which was confirmed after observing hundreds of colonies of *E. coli* growing on an LB plate containing both KANA and TMP antibiotics. The expression of *dfrB10* was induced by IPTG (isopropyl β-D-1-thiogalactopyranoside; Fig. 1). This result shows that the recombinant pET24a-*dfrB10* containing *E. coli* resists TMP because of the expression of the *dfrB10* gene.

### Construction of *dfrB10-*based cloning plasmid pUC19(TmpR)

**Fig 2:**
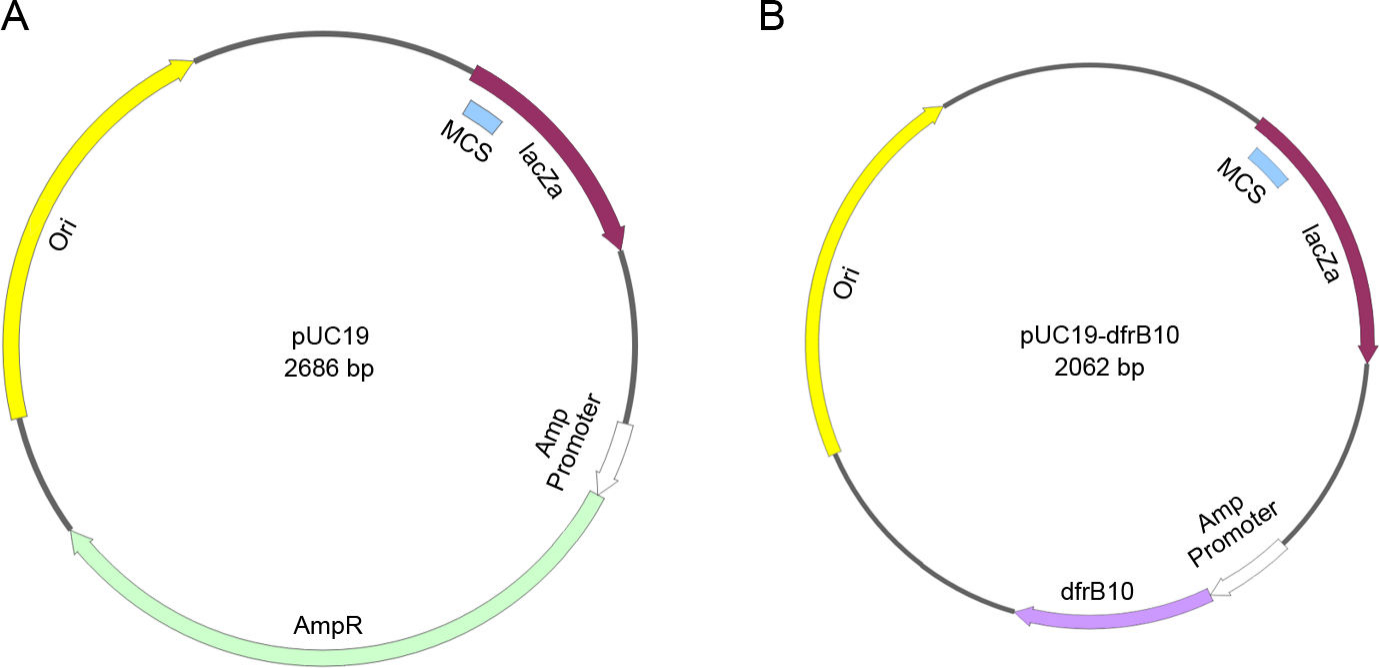
The map of cloning plasmid pUC19(TmpR). (**A**) Map of pUC19, which is AMP resistance. (**B**) Map of pUC19(TmpR), which is derived from the pUC19. The AmpR gene was substituted with TmpR (*dfrB10*).

The AmpR gene on the pUC19 backbone was removed via PCR amplification. The resulting PCR product was ligated with the *dfrB10* fragment, which was then inserted downstream of AmpR promoter. Transformation results confirmed that the resulting pUC19(TmpR) plasmid containing *E. coli* (DH5α) could grow on an LB plate with TMP (50 mg/L) antibiotic. Sequencing results showed that recombinant pUC19(TmpR) was constructed by substituting AmpR with TmpR (Fig. 2).

#### The resistance of pUC19(TmpR) recombinant *E. coli* against TMP

**Fig 3:**
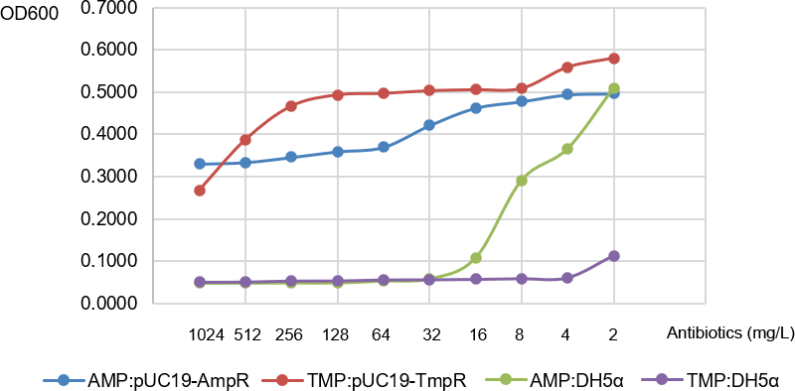
The growth results of *E. coli* DH5α bearing pUC19(TmpR). *E. coli* DH5α bearing pUC19(TmpR) plasmid could grow in the wells containing 8–128 mg/L TMP, and empty DH5α could be inhibited starting from 4 mg/L.

The microplate method was used to test the resistance against TMP of *E. coli* DH5α, which was bought by plasmid pUC19(TmpR). After 12 hours of growth at 37°C, turbidity results showed that *E. coli* DH5α carrying pUC19(TmpR) plasmid could grow in the wells containing antibiotic TMP ranging from 8 to 128 mg/L. In contrast, the empty *E. coli* DH5α could not grow in the wells under the same drug concentration (Fig. 3). A working concentration of 64 mg/L was chosen for TMP selection in subsequent experiments, as positive bacteria grew normally at this concentration while the growth of non-recombinant bacteria was inhibited.

### Application of *dfrB10-*based cloning plasmid pUC19(TmpR)

**Fig 4:**
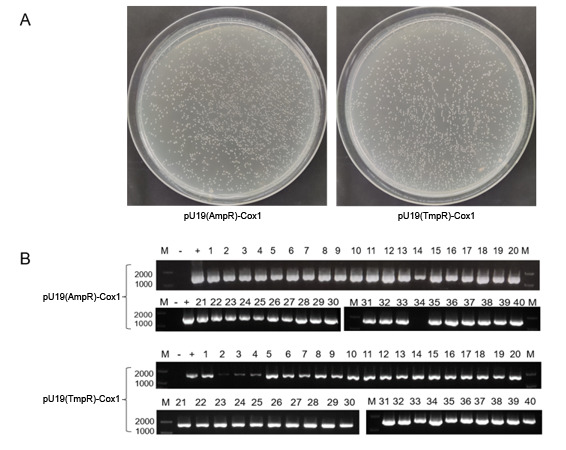
The comparison of cloning gene by using plasmid pUC19(TmpR) or pUC19(AmpR). (**A**) The colony growth results of transformation by two plasmids. The numbers of colonies were similar on the LB plate with AMP and TMP. (**B**) Colony PCR results of cloning by two plasmid vectors.

Under the same conditions, the TMP- and AMP-resistant pUC19 plasmids were used to clone a 1,536-bp gene (*Cox1*) of *Procambarus clarkii*. After ligation and transformation separately, recombinant colonies were grown on the LB plates containing TMP or AMP, and the colony densities on the plates with the different drugs were found to be similar (Fig. 4A). Forty colonies were picked from each AMP and TMP plate and their colony PCR results showed that 39 colonies were positive for AMP group (97.5%, 39/40) and 40 colonies were positive for TMP group (100.0%, 40/40). Data were analyzed using the paired samples *t* test, and there was no significant difference (*P* > 0.05) in the positive rates for cloning gene by using plasmid pUC19(TmpR) or pUC19(AmpR) (Fig. 4B).

### Construction and application of *dfrB10*-based prokaryotic expression plasmid pET28a(TmpR)

**Fig 5:**
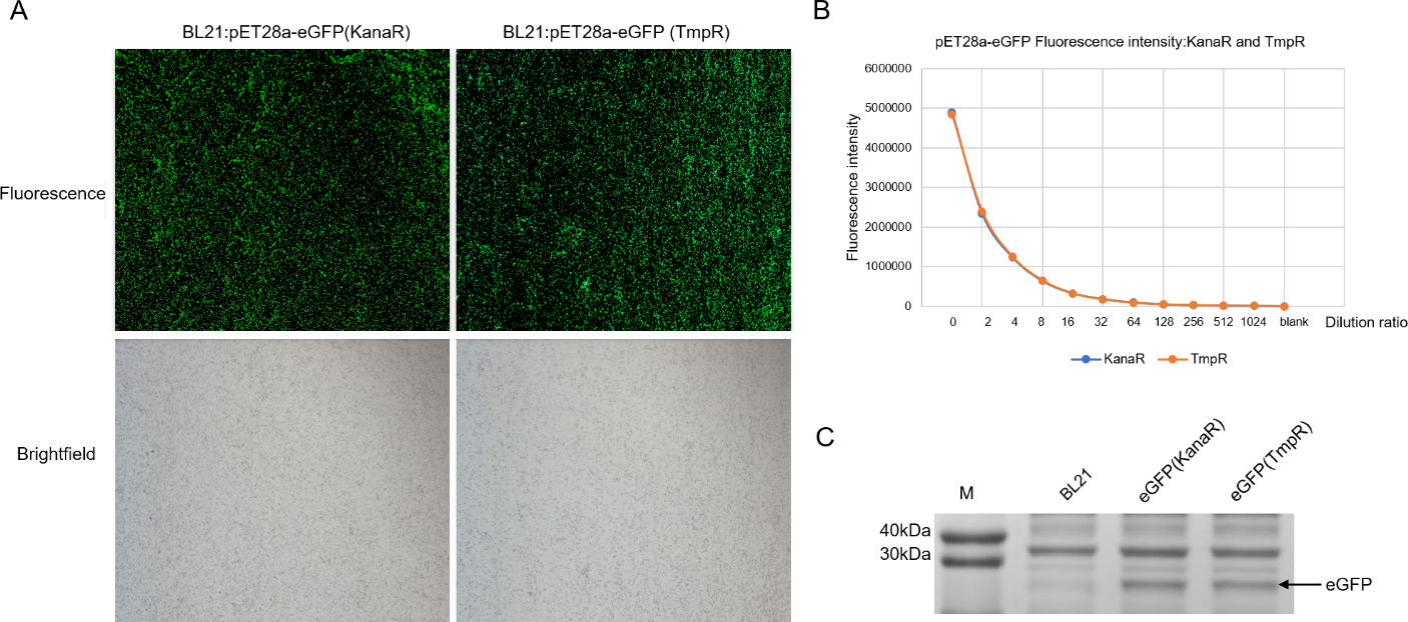
The comparison of eGFP expression of pET28a-eGFP (TmpR) and pET28a-eGFP (KanaR) in *E. coli*.

The *dfrB10-*based prokaryotic expression plasmid pET28a-eGFP (TmpR) was constructed from pET28a-eGFP (KanaR) by replacing the KanaR gene with the TmpR gene. The two plasmids were separately transformed into BL21 DE3+ and induced with IPTG. The bacterial cells were collected and observed under a fluorescence microscope, where the green fluorescence intensity was found to be similar for bacteria from both sources (Fig. 5A). The intensity of fluorescence of serial-diluted bacteria suspensions was closely related (Fig. 5B). The SDS-PAGE electrophoresis results also showed similar expression of eGFP protein in both sources of bacteria (Fig. 5C).

### Construction and application of *dfrB10*-based eukaryotic expression plasmid pcDNA3.1-eGFP (TmpR)

**Fig 6:**
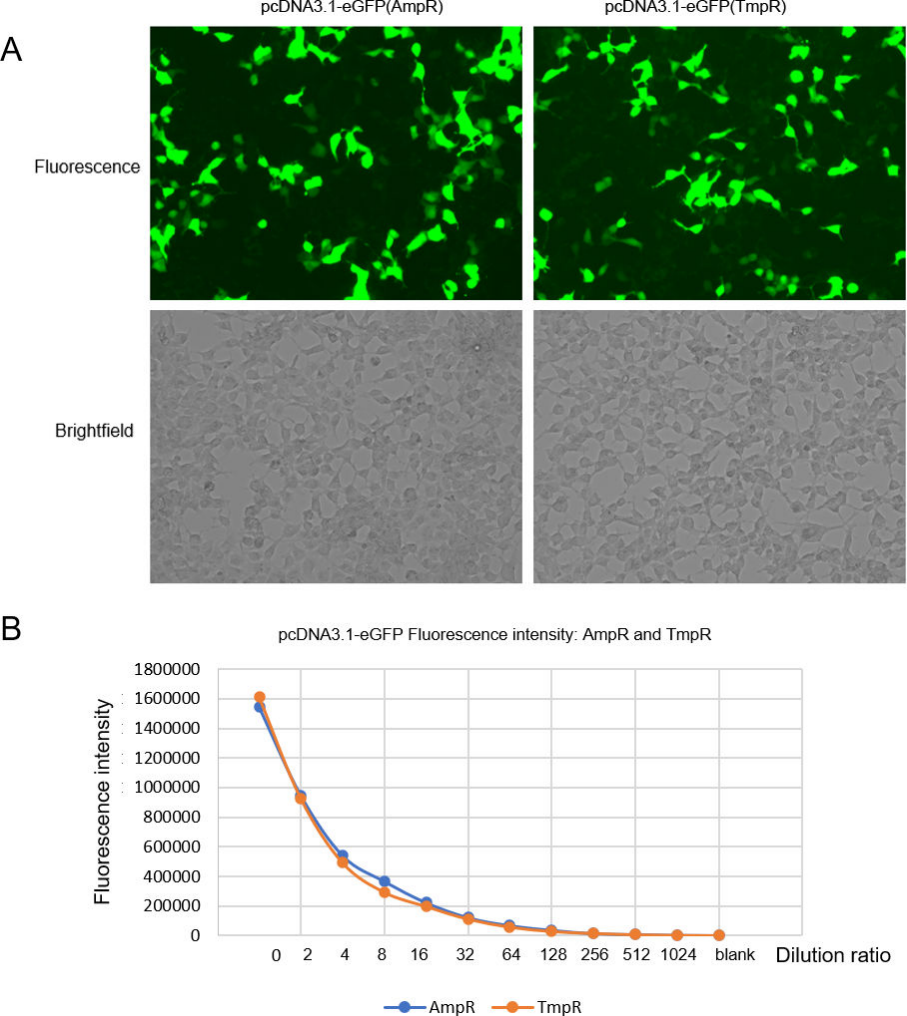
The comparison of expression of pcDNA3.1-eGFP (TmpR) and pcDNA3.1-eGFP (AmpR) in HEK293 cells.

The AmpR gene on pcDNA3.1-eGFP plasmid was replaced with TmpR gene, resulting in the construction of *dfrB10*-based eukaryotic expression plasmid pcDNA3.1-eGFP (TmpR). HEK293 cells were transfected with either pcDNA3.1-eGFP (TmpR) or the original plasmid pcDNA3.1-eGFP (AmpR) using Lipo2000. After 24 hours of culture, the green fluorescence intensity expressed by the transfected cells was observed under a fluorescence microscope. Results showed that the intensity of green fluorescence in both types of transfected cells was similar (Fig. 6).

### Construction and comparison of *dfrB10*-based CRISPR plasmids pX330-sgRNA (TmpR) and pX330-sgRNA (AmpR)

**Fig 7:**
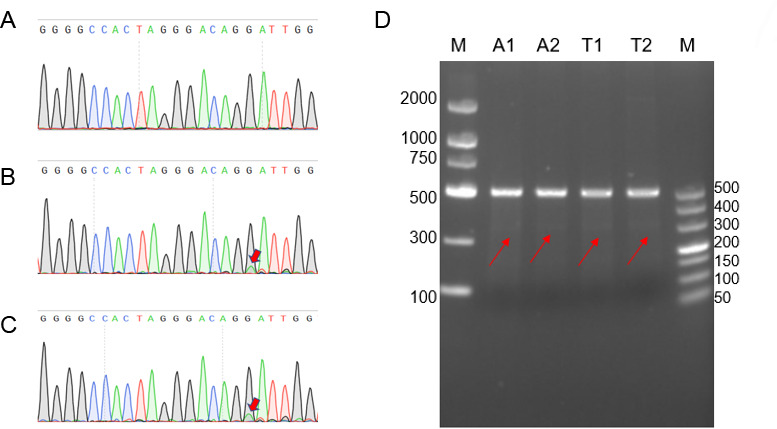
Sequencing and gel electrophoresis results of AAVS1 targeting fragment after T7E1 digestion. (**A**) Negative control. (**B**) pX330-sgRNA (AmpR) group. (**C**) pX330-sgRNA (TmpR) group. The red arrows indicated overlapped traces. (**D**) Gel electrophoresis results. A1, A2: HEK293 cells transfected with pX330-sgRNA (AmpR) plasmid; T1, T2: HEK293 cells transfected with pX330-sgRNA (TmpR) plasmid, the red arrow indicates the 299 bp bands.

Two CRISPR plasmids, pX330-sgRNA (AmpR) and pX330-sgRNA (TmpR), were successfully constructed to target the AAVS1 gene using the same crRNA sequence. HEK293 cells were separately transfected with each plasmid, and after 72 hours, the cells were collected for genomic DNA extraction. PCR amplification with primer CEI-F/R followed by sequencing revealed that both plasmids induced similar mutations. The PCR product was also digested with enzyme T7E1, and gel analysis showed that both plasmids generated similar hetero fragments (Fig. 7).

### Construction of the minimized tool plasmid pTMi with TMP resistance

**Fig 8:**
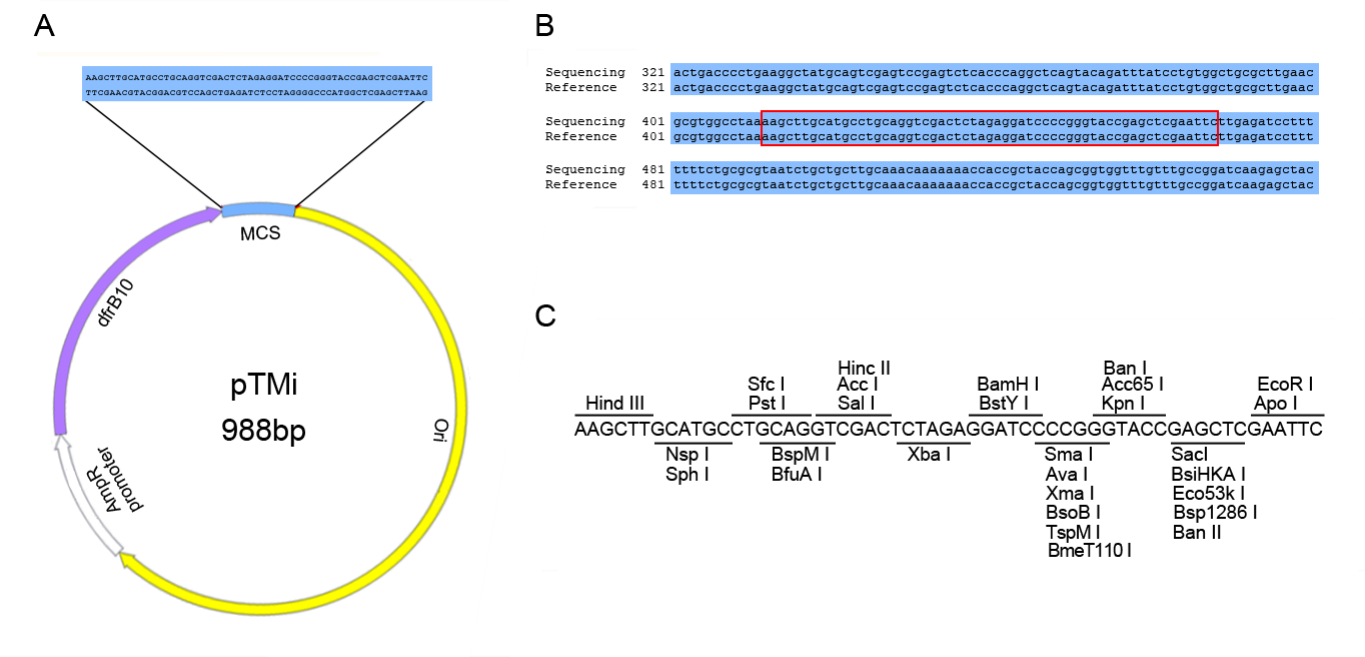
The construction of minimized tool plasmid pTMi. (**A**) The map of pTMi. (**B**) The sequencing result of MCS of pTMi. (**C**) Restriction enzyme sites in the MCS.

A minimized tool plasmid pTMi was successfully constructed, which is only 988 bp in length and might be the smallest artificially constructed cloning vector reported so far. The pTMi consisted of only three seamless parts: a 589-bp pUC origin, a 342-bp promoter-*dfrB10* drug selection marker, and a 57-bp MCS sequence (GenBank accession: OR065072). This small plasmid can self-replicate in *E. coli* and has TMP resistance (Fig. 8). Small, medium, and large fragment cloning experiments using pTMi have all yielded positive results indicating that pTMi is suitable for both classical double digestion methods and more convenient homologous recombination.

### The copy number and the stability of *dfrB10*-based plasmids

qPCR was used to determine the copy number of *dfrB10*-based plasmids pTMi and pUC19(TmpR) in *E. coli*. The result showed that there were 620 copies of pTMi plasmids per DH5α cell. For pUC19(TmpR), there were 560 copies per cell. pUC19(TmpR) containing *E. coli* DH5α were continuously cultured in antibiotic-free broth for 10 days. On the last day, there were many clones growing on the TMP-LB plate, and the retention rate of the pUC19(TmpR) plasmid was 39.0%.

## DISCUSSION

In the late 1950s, several research groups started studying plasmids after discovering that extrachromosomal antibiotic resistance (R) factors are responsible for the transmissibility of multiple antibiotic resistance among the enterobacteria (17). The R factor, also known as the drug resistance factor, is the plasmid that makes bacteria resistant to antibiotics. It carries genes that encode certain enzymes that can inactivate streptomycin, chloramphenicol, and sulfa drugs (18). Plasmids can carry more than one drug-resistant gene, which are called multidrug resistance factors. The spread of drug-resistant bacteria poses a serious threat to human health (19). However, plasmid-mediated antibiotic resistance can also be used for a variety of molecular genetic studies. Currently, various antibiotics and corresponding resistance genes are used as drug selection markers in plasmid construction. Common drug-resistant genes carried by plasmids include tetracycline, penicillin, chloramphenicol, and streptomycin (20). The mechanisms behind drug resistance include the destruction or modification of antibacterial agents, blocking of antibacterial agents from reaching their targets in bacterial cells, or producing changed bacterial targets. The length of resistant genes is typically more than 600 bp. The reported smallest plasmid, pICOz (1,185 bp), utilizes the resistance gene *Sh ble* (375 bp), which can resist the inhibitory effect of Zeocin, an antibiotic belonging to the phleomycin family (21, 22). Using the Zeocin resistance gene can reduce the plasmid size by about 400 bp compared to using AmpR or KanaR.

The TMP resistance gene, *dfrB10*, belongs to the *dfrB* family, and its coding gene is only 237 bp. Its product, as a subunit, can constitute a homotetrameric enzyme protein that has dioxyfolate-reducing function (23). Because of the difference in conformation of its active center, the *dfrB10* product has a low binding force to TMP (11, 24). By expressing the *dfrB10* gene through the plasmid, bacteria can acquire additional dioxyfolate-reducing ability. In the presence of TMP, it can counteract the inhibition of the drug on the host’s own folic acid metabolism pathway so that the recipient bacteria can survive. TMP and *dfrB10* together constitute a shorter antibiotic selection marker system that is suitable for miniature plasmid construction.

Antibiotics are crucial in the plasmid biological selection marker system, and they must be carefully considered when constructing plasmids. AMP and KANA are commonly used antibiotics in clinical settings due to their stability and easy availability, making them practical options. In contrast, although the Zeocin- resistance gene is small, the cost of the antibiotic Zeocin is prohibitively high, particularly for large-scale expression, making it an unsuitable choice. TMP is a widely used synthetic antibiotic that is structurally similar to folic acid (25). It acts as a competitive inhibitor of bacterial DHFR. The TMP powder used in this study can be dissolved in dimethyl sulfoxide and prepared as a 64 mg/mL TMP stock solution. The application method for TMP in preparing plates and liquid culture media is similar to AMP and KANA. TMP is also cheaper, highly stable, and easy to use (26). Our study found that TMP has a stronger antibacterial effect than AMP on non-resistant *E. coli* at low concentrations, but at higher concentrations, the inhibitory effect on recombinant *E. coli* carrying resistant plasmids was weaker, resulting in a high OD600 value. Currently, TmpR-based plasmids are not tested for gene therapy, but these results suggest that TMP may be a useful antibiotic selection marker for recombinant plasmids, with certain advantages. Moreover, TMP is generally less allergic than AMP as a drug, using *dfrB10*-based plasmid to produce recombinant protein, and the antibiotic residual risk would be considerable low. TmpR-based plasmid itself may also be less allergic due to its smaller size. Thus, TMP-*dfrB10* could play an important role in the preclinical research of vaccine or gene therapy.

This study successfully generated a series of functional plasmids based on the TMP-*dfrB10* gene, including cloning, prokaryotic and eukaryotic expression, and CRISPR plasmids. Preliminary results showed that the efficiency of the TMP-based cloning plasmid pUC19(TmpR) was comparable to the original pUC19(AmpR) plasmid for cloning PCR products. For proteins that are difficult to express in *E. coli*, engineering bacteria like Rosetta(DE3+) carrying plasmids with rare codons can increase the likelihood of successful expression (27). If both plasmids use the same resistance selection marker, it can impact the selection of protein expression plasmids. Prokaryotic expression of pET28a-eGFP (TmpR) showed that using TMP as an antibiotic selection marker did not affect eGFP expression, indicating that TMP plasmids could be used as co-expression plasmids. Furthermore, TMP-based pcDNA3.1 plasmid could express fluorescence normally in recipient cells, and there were no significant differences in fluorescence expression compared to the original KANA-based plasmid. This suggested that changing the drug screening marker to TMP would not affect the expression of foreign genes in prokaryotic and eukaryotic cells. Genome editing on HEK293 cells further demonstrated that the TMP-based CRISPR plasmid had similar gene editing capabilities to the original plasmid. The design of the plasmid used in this study is similar to the design of the pminiR1 plasmid, and both use the AmpR promoter to drive non-AMP resistance gene as a drug selection marker (10, 28). Different from the pUC replicon used in this study, the pminiR1 plasmid is based on the R1 replicon, and the new plasmid containing only the minimal elements was constructed by synthetic method. In this study, the strategy for constructing size-reduced plasmids is modifying classic plasmids, and the small-sized *dfrB10* resistance gene was used to replace the larger original resistance selection marker to achieve the goal.

In this study, it was found that changing the screening marker to TMP resulted in a reduction of the plasmid size by 624 bp or 579 bp when compared to AMP- or KANA-based plasmids, respectively. The Ori, prokaryotic promoter, and the resistance gene *dfrB10* were directly ligated through seamless cloning, resulting in a 988-bp plasmid, which is 197 bp smaller than the current smallest tool plasmid pICOz. Using more compact genes to reduce plasmid size without affecting its function may benefit various researches in molecular biology. Moreover, the smaller plasmid size may offer advantages in the transfection of eukaryotic cells and may enrich the genetic edition tool box for various organisms. However, the cloning efficiency is influenced by the vector and insert fragment’s ratio and quality, and various factors such as growth, induction, yield, and cost must be balanced to obtain optimal gene expression efficiency. Future research needs to verify whether TMP is universally applicable to the expression of foreign proteins, particularly in research related to gene editing and gene therapy.

This study successfully replaced resistance genes on various tool plasmids with shorter dihydrofolate reductase genes, creating a series of tool plasmids with TMP resistance. Preliminary studies showed that these new size-reduced plasmids functioned similarly to the original plasmids, providing a new tool for molecular genetic manipulation. Additionally, the study created the smallest tool plasmid to date, which has significant potential for future research.

## MATERIALS AND METHODS

### Bacterial strains, cell lines, and plasmids

DH5α, BL21 DE3+ competent cells and plasmid extraction kit were purchased from Tiangen (Beijing, China). Plasmids pUC19, pET28a-eGFP, pcDNA3.1-eGFP, pX330, and HEK293 cell line were purchased from Miaoling Biosystem (Wuhan, China). PCR primer sequences were synthesized by Genscript Biotechnology (Nanjing, China).

### Obtaining of *dfrB10* gene and verification of its TMP resistance function

The *dfrB10* gene sequence was retrieved from Genbank under access number KU130294, in which 208178–208414 (237 bp) is the CDS of *dfrB10* gene (23, 29). The synthesized *dfrB10* fragment was obtained from Genscript Biotechnology and was inserted into the cloning site of pET24a vector to construct the expression plasmid of pET24a-*dfrB10*. The *E. coli* strain BL21 DE3+ was transformed with the pET24a-*dfrB10* plasmid using the heat shock method, and the transformed bacteria was spread on LB plates containing both KANA (50 mg/L) and TMP (50 mg/L) antibiotics (30). TMP was purchased from Aladdin (Shanghai, China). Empty pET24a plasmid was used as the negative and the positive control (with or without TMP). All LB plates were supplemented with IPTG (0.1 mmol/L) and incubated at 37°C overnight (12–14 hours). The growth of the colonies was observed the next day.

### The construction of *dfrB10-*based cloning plasmid pUC19(TmpR)

Primers for amplification of *dfrB10* and pUC19 backbone (without AmpR, retaining the AmpR promoter) were designed using CE Design software (Vazyme, Nanjing, China). The Tks Gflex DNA Polymerase (Takara, Otsu, Japan) was used to amplify the pUC19 backbone and the *dfrB10* fragment (31). The ClonExpress Ultra One Step Cloning Kit (Vazyme, Nanjing, China) was used to ligate fragments of *dfrB10* and pUC19 backbone (32). All experiments were conducted according to the manufacturer’s instructions, and primers and reaction conditions are shown in Table 1. We used 10 µL of the ligation product to transform *E. coli* DH5α competent cells and screen recombinant clone on LB plate with TMP (50 mg/L). Single colonies were picked and cultured in LB broth with shaking, and then the samples were sent to Sangon Biotech (Shanghai, China) for sequencing. The plasmid with the correct *dfrB10* sequence was named pUC19(TmpR).

**TABLE 1:** Primers used in the present study

Primer name^*a*^	Sequence (5'−3')	Reacting conditions							
pUC19-F	CTGTCAGACCAAGTTTACTCATATATACTTT	94°C, 1 min 98°C, 10 s 50°C, 15 s 68°C, 1 min (2–4, 30 cycles) 68°C, 10 min							
pUC19-R	ACTCTTCCTTTTTCAATATTATTGAAGC								
19-*dfrB10-*F	AATATTGAAAAAGGAAGAGTATGGATCAAAGTAGCAATGAAGTCA	94°C, 1 min 98°C, 10 s 55°C, 15 s 68°C, 15 s (2–4, 30 cycles) 68°C, 10 min							
19-*dfrB10-*R	GAGTAAACTTGGTCTGACAGTTAGGCCACGCGTTCAAGC								
**COX1-F**	GAGCTCGGTACCCGGGGATCCACGCAACGATGATTTTTTTCTACA	94°C, 1 min 98°C, 10 s 55°C, 15 s 68°C, 1 min (2–4, 30 cycles) 68°C, 10 min							
**COX1-R**	GACCATGATTACGCCAAGCTTTTAATTAGAAATTAATGGAATCTCAGAATAA								
pET28a-eGFP-F	GAATTAATTCATGAGCGGATACATATT	94°C, 1 min 98°C, 10 s 55°C, 15 s 68°C, 3 min (2–4, 30 cycles) 68°C, 10 min							
pET28a-eGFP-R	AACACCCCTTGTATTACTGTTTATGTAAG								
28-*dfrB10-*F	ACAGTAATACAAGGGGTGTTATGGATCAAAGTAGCAATGAAGTCA	94°C, 1 min 98°C, 10 s 55°C, 15 s 68°C, 15 s (2–4, 30 cycles) 68°C, 10 min							
28-*dfrB10-*R	ATCCGCTCATGAATTAATTCTTAGGCCACGCGTTCAAGC								
pCDNA3. 1-eGFP-F	CTGTCAGACCAAGTTTACTCATATATACTTT	94°C, 1 min 98°C, 10 s 50°C, 15 s 68°C, 5 min (2–4, 30 cycles) 68°C, 10 min							
pCDNA3. 1-eGFP-R	ACTCTTCCTTTTTCAATATTATTGAAGC								
1-*dfrB10-*F	AATATTGAAAAAGGAAGAGTATGGATCAAAGTAGCAATGAAGTCA	94°C, 1 min 98°C, 10 s 55°C, 15 s 68°C, 15 s (2–4, 30 cycles) 68°C, 10 min							
1-*dfrB10-*R	GAGTAAACTTGGTCTGACAGTTAGGCCACGCGTTCAAGC								
pX330-F	CTGTCAGACCAAGTTTACTCATATATACTTT	94°C, 1 min 98°C, 10 s 50°C, 15 s 68°C, 5 min (2–4, 30 cycles) 68°C, 10 min							
pX330-R	ACTCTTCCTTTTTCAATATTATTGAAGC								
330-*dfrB10-*F	AATATTGAAAAAGGAAGAGTATGGATCAAAGTAGCAATGAAGTCA	94°C, 1 min 98°C, 10 s 55°C, 15 s 68°C, 15 s (2–4, 30 cycles) 68°C, 10 min							
330-*dfrB10-*R	GAGTAAACTTGGTCTGACAGTTAGGCCACGCGTTCAAGC								
AAVS1-sgRNA-F^*^	CCACGGGCCACTAGGGACAGGAT	95°C	75°C	60°C	55°C	40°C	25°C	6°C	4°C
AAVS1-sgRNA-R^*^	AAACATCCTGTCCCTAGTGGCCC	30 s	30 s	30 s	30 s	30 s	30 s	30 s	1h
CEI-F^*^	TTCGGGTCACCTCTCACTCC	94°C, 1 min 98°C, 10 s 50°C, 15 s 68°C, 40 s (2–4, 30 cycles) 68°C, 10 min							
CEI-R^*^	GGCTCCATCGTAAGCAAACC								
Ori-F	TTGAGATCCTTTTTTTCTGCGC	94°C, 1 min 98°C, 10 s 50°C, 15 s 68°C, 45 s (2–4, 30 cycles) 68°C, 10 min							
Ori-R	TTTCCATAGGCTCCGCCC								
AMP-promoter-F	GGGGGCGGAGCCTATGGAAACGCGGAACCCCTATTTGTT	94°C, 1 min 98°C, 10 s 55°C, 15 s 68°C, 20 s (2–4, 30 cycles) 68°C, 10 min							
*dfrB10-*R	GCAGAAAAAAAGGATCTCAATTAGGCCACGCGTTCAAGC								

^*a*^ Primers marked with an asterisk were designed by Yu et al. (33), and other primers were designed during this study.

### Detection of TMP resistance concentration of recombinant bacteria based on *dfrB10*

pUC19(TmpR) plasmid containing DH5α strain was used to determine the TMP resistance of the recombinant bacteria. Following the method recommended by the American Clinical Laboratory Standardization Institute (34), we adjusted the bacterial suspension to a turbidity equivalent to a 0.5 McFarland standard (1.5 × 10^8^ CFU/mL). TMP solution was diluted 10-fold by LB broth from columns 3 to 12 (1,024, ~2 mg/L) on the 96-well plates, and 100 µL bacterial suspension was added to each well. The wells of column A were used as the blank and 200 µL of LB broth was added. The test plates were incubated with shaking for 12 hours at 37°C, and OD600 was measured. Bacteria containing original pUC19(AmpR) plasmid and empty DH5α were used as controls; each concentration had six duplicates.

### Application of *dfrB10-*based cloning plasmid pUC19(TmpR)

pUC19(TmpR) and pUC19(AmpR) were digested by *BamHI* and *HindIII* (NEB, Beijing, China), respectively, and the vector fragments were recovered after digestion. A 1,536-bp gene of *P. clarkii* (Genbank: NC_016926) was selected and PCR amplified as the insert fragment. ClonExpress Ultra One Step Cloning Kit was used to conjugate the vector and insert. To compare the cloning efficiency of the two plasmids, we set the DNA concentrations and molar ratios of the two vectors and inserts as per the protocol. After ligation, the product was spread on a LB plate supplemented with AMP (100 mg/L) or TMP (64 mg/L), and the working concentration was determined as above. Forty colonies were picked and identified by PCR after overnight culture. SPSS 13.0 software was used for statistical analysis. The primer sequences and PCR conditions are shown in Table 1.

### The *dfrB10*-based prokaryotic expression plasmid pET28a(TmpR) and its application

Prokaryotic expression plasmid pET28a-eGFP (TmpR) was constructed by replacing the KanaR gene with *dfrB10* gene. The cloning method is similar to the construction pUC19(TmpR), and the primer sequences and PCR conditions are shown in Table 1. Both the plasmids were transformed into BL21 DE3+, and selection was done using IPTG-pretreated TMP and KANA plates. Then, the fluorescent expression colonies were picked, cultured, and induced with IPTG with shaking at 37°C. Fluorescent expression was observed and compared using a fluorescence microscope, and eGFP expression was analyzed using SDS-PAGE electrophoresis. The fluorescence intensity was measured using a microplate reader after serial dilution of both bacterial cultures.

### The *dfrB10-*based eukaryotic expression plasmid pcDNA3. 1(TmpR) and its application

The *dfrB10*-based eukaryotic expression plasmid pcDNA3. 1(TmpR) was constructed by replacing the AmpR gene with *dfrB10* gene, and its effectiveness was compared with pcDNA3. 1-eGFP (AmpR) in terms of eGFP expression in HEK293 cells. The cloning and screening methods are the same as above, and the primer sequences and PCR conditions are shown in Table 1. The plasmids were transfected into cells using the EZ *Trans* reagent (Life iLab Biotech, Shanghai, China) (35), and the expression of eGFP was observed and compared using a microscope. The fluorescence intensity was measured using a microplate reader after dilution.

### The *dfrB10-*based CRISPR plasmid pX330(TmpR) and its application

CRISPR plasmid pX330(TmpR) was constructed by replacing the AmpR gene with *dfrB10* gene. The AAVS1 gene of HEK293 cells was selected as the gene editing target. sgRNA oligos were synthesized by Genscript, and after annealing, the double-strand DNA fragments were inserted into CRISPR plasmids. The cloning and screening method used was the same as above. The primer, oligo sequences, and PCR conditions are listed in Table 1. The TMP- and AMP-resistant pX330 plasmids were separately transfected into HEK293 cells according to the protocol of the reagent Lipofectamine 2000 (Thermo Fisher, Waltham, USA) (36). After 72 hours of culture, the cells were collected, and the genomic DNA of the cells was extracted. T7E1 enzyme (Vazyme, Nanjing, China) was used to digest the insertions and mutations introduced by NHEJ repair after CRISPR/Cas9 editing (37). At the same time, the PCR product of AAVS1 gene was sequenced by Sangon Biotech, and the tracing file was analyzed for mutations.

### The construction of *dfrB10-*based minimal tool plasmid pTMi (minimized plasmid with TMP resistance)

The construction of *dfrB10*-based minimal tool plasmid pTMi (minimized plasmid with TMP resistance) was done by PCR amplifying the Ori and TmpR fragments from pUC19(TmpR) and joining them using the ClonExpress Ultra One Step Cloning Kit. Transformation and screening were performed according to the method mentioned above. The MCS sequence was added to the backbone of the new vector by PCR and homologous recombinant. The TMP resistance colonies were picked and cultured, and subsequently sent to Sangon Biotech for sequencing. The minimized plasmid with the expected sequence was named pTMi.

### The copy number and the stability of *dfrB10*-based plasmids

Overnight cultures of pTMi and pUC19(AmpR) plasmid *E. coli* were adjusted to 4.8 × 10^8^ cells/mL, then 1:10,000 diluted. The samples were lysed under 95°C for 20 min. After 13,400 rpm/min spin for 10 min, the supernatant was collected as a qPCR template. A pair of primer targeting the pUC origin were selected, and purified pUC origin containing plasmid pET28a-eGFP (29.7 µg/µL, equals 4. 78 × 10^9^ copies/μL) were serial diluted as standards (from 10^−2^ to 10^−6^). The 20-µL reaction mixture contained 10.0 µL of 2× SYBR Green qPCR Mix (Biosharp, China), 0.5 µL of each primer, 0.5 µL of ROX, 1 µL of the template, and 7.5 µL of DDW. All qPCR reactions were conducted in triplicate on the Long Gene (Hangzhou, China), and the conditions were as follows: 95°C for 30 s and then 60°C for 1 min, for 40 cycles. Copy numbers were calculated according to Chen et al. (38).

pUC19(TmpR) containing *E. coli* DH5α was overnight cultured in TMP-LB broth under 37°C with 200 rpm/min shaking. The old culture was 1:1,000 diluted and cultured in drug-free LB broth for 10 days, and temperature and shaking conditions were the same. On the last day, the culture was 1:2,000 diluted and spread on the drug-free and TMP-LB plates. The retention rate of plasmids was calculated by dividing the number of colonies on TMP-LB plate by the number of colonies on antibiotic-free LB plate (39).

## References

[1] Rouches MV , Xu Y , Cortes LBG , Lambert G . 2022. A plasmid system with tunable copy number. Nat Commun 13:3908. doi:10.1038/s41467-022-31422-0 35798738 PMC9263177

[2] James S , Jain V . 2021. A positive selection Escherichia coli recombinant protein expression vector for one-step cloning. Front Bioeng Biotechnol 9:776828. doi:10.3389/fbioe.2021.776828 35047486 PMC8761972

[3] Hall JPJ , Botelho J , Cazares A , Baltrus DA . 2022. What makes a megaplasmid ? Philos Trans R Soc Lond B Biol Sci 377:20200472. doi:10.1098/rstb.2020.0472 34839707 PMC8628078

[4] Kreiss P , Cameron B , Rangara R , Mailhe P , Aguerre-Charriol O , Airiau M , Scherman D , Crouzet J , Pitard B . 1999. Plasmid DNA size does not affect the physicochemical properties of lipoplexes but modulates gene transfer efficiency. Nucleic Acids Res 27:3792–3798. doi:10.1093/nar/27.19.3792 10481017 PMC148641

[5] Staal J , Alci K , De Schamphelaire W , Vanhoucke M , Beyaert R . 2019. Engineering a minimal cloning vector from a pUC18 Plasmid backbone with an extended multiple cloning site. Biotechniques 66:254–259. doi:10.2144/btn-2019-0014 31124712

[6] Lesueur LL , Mir LM , André FM . 2016. Overcoming the specific toxicity of large plasmids electrotransfer in primary cells in vitro. Mol Ther Nucleic Acids 5:e291. doi:10.1038/mtna.2016.4 27111417 PMC5014460

[7] Schnödt M , Schmeer M , Kracher B , Krüsemann C , Espinosa LE , Grünert A , Fuchsluger T , Rischmüller A , Schleef M , Büning H . 2016. DNA minicircle technology improves purity of adeno-associated viral vector preparations. Mol Ther Nucleic Acids 5:e355. doi:10.1038/mtna.2016.60 28131313 PMC5023404

[8] Williams JA , Paez PA . 2023. Improving cell and gene therapy safety and performance using next-generation nanoplasmid vectors. Mol Ther Nucleic Acids 32:494–503. doi:10.1016/j.omtn.2023.04.003 37346980 PMC10280095

[9] Then RL . 2004. Antimicrobial dihydrofolate reductase inhibitors--achievements and future options: review. J Chemother 16:3–12. doi:10.1179/joc.2004.16.1.3 15077993

[10] Islas F , Sabido A , Sigala J-C , Lara AR . 2023. Design of microaerobically inducible miniR1 plasmids. mLife 2:101–104. doi:10.1002/mlf2.12058 PMC10989972 38818336

[11] Cammarata M , Thyer R , Lombardo M , Anderson A , Wright D , Ellington A , Brodbelt JS . 2017. Characterization of trimethoprim resistant E. coli dihydrofolate reductase mutants by mass spectrometry and inhibition by propargyl-linked antifolates. Chem Sci 8:4062–4072. doi:10.1039/c6sc05235e 29967675 PMC6020862

[12] Fleming MP , Datta N , Grüneberg RN . 1972. Trimethoprim resistance determined by R factors. Br Med J 1:726–728. doi:10.1136/bmj.1.5802.726 4552466 PMC1787613

[13] Lemay-St-Denis C , Diwan SS , Pelletier JN . 2021. The bacterial genomic context of highly trimethoprim-resistant DfrB dihydrofolate reductases highlights an emerging threat to public health. Antibiotics (Basel) 10:433. doi:10.3390/antibiotics10040433 33924456 PMC8103504

[14] Sun F , Zhou D , Wang Q , Feng J , Feng W , Luo W , Liu Y , Qiu X , Yin Z , Xia P . 2016. Genetic characterization of a novel bla_DIM-2_-carrying megaplasmid p12969-DIM from clinical Pseudomonas putida. J Antimicrob Chemother 71:909–912. doi:10.1093/jac/dkv426 26679251

[15] Sánchez-Osuna M , Cortés P , Llagostera M , Barbé J , Erill I . 2020. Exploration into the origins and mobilization of di-hydrofolate reductase genes and the emergence of clinical resistance to trimethoprim. Microb Genom 6:mgen000440. doi:10.1099/mgen.0.000440 32969787 PMC7725336

[16] Alonso H , Gready JE . 2006. Integron-sequestered dihydrofolate reductase: a recently redeployed enzyme. Trends Microbiol 14:236–242. doi:10.1016/j.tim.2006.03.003 16584884

[17] Helinski DR . 2022. A brief history of plasmids. EcoSal Plus 10:eESP00282021. doi:10.1128/ecosalplus.esp-0028-2021 35373578 PMC10729939

[18] Abushaheen MA , Muzaheed , Fatani AJ , Alosaimi M , Mansy W , George M , Acharya S , Rathod S , Divakar DD , Jhugroo C , Vellappally S , Khan AA , Shaik J , Jhugroo P . 2020. Antimicrobial resistance, mechanisms and its clinical significance. Dis Mon 66:100971. doi:10.1016/j.disamonth.2020.100971 32201008

[19] Wang Z , Koirala B , Hernandez Y , Zimmerman M , Park S , Perlin DS , Brady SF . 2022. A naturally inspired antibiotic to target multidrug-resistant pathogens. Nature 601:606–611. doi:10.1038/s41586-021-04264-x 34987225 PMC10321319

[20] Funnell BE , Phillips GJ . 2004. Plasmid biology. ASM Press, Washington, DC, USA. doi:10.1128/9781555817732

[21] Bennett RP , Cox CA , Hoeffler JP . 1998. Fusion of green fluorescent protein with the Zeocin-resistance marker allows visual screening and drug selection of transfected eukaryotic cells. Biotechniques 24:478–482. doi:10.2144/98243pf01 9526661

[22] Hudecova A , Hasplova K , Kellovska L , Ikreniova M , Miadokova E , Galova E , Horvathova E , Vaculcikova D , Gregan F , Dusinska M . 2012. Gentiana asclepiadea and Armoracia rusticana can modulate the adaptive response induced by zeocin in human lymphocytes. Neoplasma 59:62–69. doi:10.4149/neo_2012_01_008 22103898

[23] Lemay-St-Denis C , Diwan SS , Pelletier JN . 2021. The bacterial genomic context of highly trimethoprim-resistant DfrB dihydrofolate reductases highlights an emerging threat to public health. Antibiotics (Basel) 10:433. doi:10.3390/antibiotics10040433 33924456 PMC8103504

[24] Strader MB , Chopra S , Jackson M , Smiley RD , Stinnett L , Wu J , Howell EE . 2004. Defining the binding site of homotetrameric R67 dihydrofolate reductase and correlating binding enthalpy with catalysis. Biochemistry 43:7403–7412. doi:10.1021/bi049646k 15182183

[25] Li WM , Xu H . 2021. Characterization and biochemistry analysis of a novel trimethoprim resistant Dfrb7 and the mechanism of trimethoprim resistance. Acta Microbiologica Sinica 61:4097–4105. doi:10.13343/j.cnki.wsxb.20210179

[26] Traub WH , Leonhard B . 1995. Heat stability of the antimicrobial activity of sixty-two antibacterial agents. J Antimicrob Chemother 35:149–154. doi:10.1093/jac/35.1.149 7768762

[27] Kwon CW , Chung B , Yoo SH , Chang PS . 2022. Heterologous expression of a papain-like protease inhibitor (SnuCalCpI17) in the E. coli and its mode of inhibition. Appl Microbiol Biotechnol 106:4563–4574. doi:10.1007/s00253-022-12032-8 35748913

[28] Lara AR , Velázquez D , Penella I , Islas F , González-De la Rosa CH , Sigala J-C . 2019. Design of a synthetic miniR1 plasmid and its production by engineered Escherichia coli *.* Bioprocess Biosyst Eng 42:1391–1397. doi:10.1007/s00449-019-02129-2 31006041

[29] Sun F , Zhou D , Wang Q , Feng J , Feng W , Luo W , Liu Y , Qiu X , Yin Z , Xia P . 2016. Genetic characterization of a novel bla_DIM-2_-carrying megaplasmid p12969-DIM from clinical Pseudomonas Putida. J Antimicrob Chemother 71:909–912. doi:10.1093/jac/dkv426 26679251

[30] Rahimzadeh M , Sadeghizadeh M , Najafi F , Arab S , Mobasheri H . 2016. Impact of heat shock step on bacterial transformation efficiency. Mol Biol Res Commun 5:257–261. 28261629 PMC5326489

[31] Wang S , Tan B , Xiao L , Zeng J , Zhao X , Hong L , Li Z , Cai G , Zheng E , Gu T , Wu Z . 2022. Long non-coding RNA Gm10561 promotes Myogenesis by sponging miR-432. Epigenetics 17:2039–2055. doi:10.1080/15592294.2022.2105052 35899799 PMC9665130

[32] Wang J , Sui X , Ding Y , Fu Y , Feng X , Liu M , Zhang Y , Xian M , Zhao G . 2021. A fast and robust Iterative genome-editing method based on a rock-paper-scissors strategy. Nucleic Acids Res 49:e12–e12. doi:10.1093/nar/gkaa1141 33270888 PMC7826264

[33] Jun-Yao YU , Xiao-Lan LI , Jian-Ping Z , Tao C , Xiao-Bing Z . 2015. Co-expression of Cas9 and sgRNA in one plasmid improves targeting efficiency at the AAVS1 locus. Biotechnol Lett 26:449–453. doi:10.3969/j.issn.1009-0002.2015.04.001

[34] Espinel-Ingroff A , Barchiesi F , Cuenca-Estrella M , Pfaller MA , Rinaldi M , Rodriguez-Tudela JL , Verweij PE . 2005. International and multicenter comparison of EUCAST and CLSI M27-A2 broth Microdilution methods for testing susceptibilities of Candida spp. to fluconazole, itraconazole, posaconazole, and voriconazole. J Clin Microbiol 43:3884–3889. doi:10.1128/JCM.43.8.3884-3889.2005 16081926 PMC1233914

[35] Li Y , Wang D , Ping X , Zhang Y , Zhang T , Wang L , Jin L , Zhao W , Guo M , Shen F , Meng M , Chen X , Zheng Y , Wang J , Li D , Zhang Q , Hu C , Xu L , Ma X . 2022. Local hyperthermia therapy induces browning of white fat and treats obesity. Cell 185:949–966. doi:10.1016/j.cell.2022.02.004 35247329

[36] Lai Y , Xu X , Zhu Z , Hua Z . 2018. Highly efficient siRNA transfection in macrophages using apoptotic body-mimic ca-PS lipopolyplex. Int J Nanomedicine 13:6603–6623. doi:10.2147/IJN.S176991 30425477 PMC6205523

[37] Li N , Gou S , Wang J , Zhang Q , Huang X , Xie J , Li L , Jin Q , Ouyang Z , Chen F , Ge W , Shi H , Liang Y , Zhuang Z , Zhao X , Lian M , Ye Y , Quan L , Wu H , Lai L , Wang K . 2021. CRISPR/Cas9-mediated gene correction in newborn rabbits with hereditary tyrosinemia type I. Mol Ther 29:1001–1015. doi:10.1016/j.ymthe.2020.11.023 33221434 PMC7934638

[38] Chen S , Larsson M , Robinson RC , Chen SL . 2018. Direct and convenient measurement of plasmid stability in lab and clinical isolates of E. coli. Sci Rep 8:6056. doi:10.1038/s41598-018-23595-w 29643388 PMC5895804

[39] Gao H , Liu Y , Wang R , Wang Q , Jin L , Wang H . 2020. The transferability and evolution of NDM-1 and KPC-2 co-producing Klebsiella pneumoniae from clinical settings. EBioMedicine 51:102599. doi:10.1016/j.ebiom.2019.102599 31911273 PMC6948161

